# Deleterious role of hepatitis B virus infection in therapeutic response among patients with rheumatoid arthritis in a clinical practice setting: a case-control study

**DOI:** 10.1186/s13075-018-1548-5

**Published:** 2018-05-02

**Authors:** Yu-Lan Chen, Jian-Zi Lin, Ying-Qian Mo, Jian-Da Ma, Qian-Hua Li, Xiao-Ying Wang, Ze-Hong Yang, Tao Yan, Dong-Hui Zheng, Lie Dai

**Affiliations:** 1 Department of Rheumatology, Sun Yat-Sen Memorial Hospital, Sun Yat-Sen University, Guangzhou, 510120 People’s Republic of China; 2 Department of Radiology, Sun Yat-Sen Memorial Hospital, Sun Yat-Sen University, Guangzhou, People’s Republic of China; 3 Zhongshan School of Medicine, Sun Yat-sen University, Guangzhou, People’s Republic of China

**Keywords:** Hepatitis B virus, Rheumatoid arthritis, Radiographic progression, Clinical response

## Abstract

**Background:**

Previous studies have revealed that hepatitis B virus (HBV) infection may be associated with rheumatoid arthritis (RA), while there are no further clinical studies regarding the role of HBV infection in RA progression during disease-modifying anti-rheumatic drug (DMARD) therapy. Here, we aimed to explore the influence of HBV infection on radiographic and clinical outcomes among patients with RA in a clinical practice setting.

**Methods:**

Thirty-two consecutive patients with RA (Disease Activity Score 28-joint assessment based on C-reactive protein (DAS28-CRP) ≥2.6) with chronic HBV infection (CHB) were retrospectively recruited as the CHB group and 128 age-matched, sex-matched, and disease activity-matched contemporary patients with RA without CHB were included in the non-CHB group. Clinical data were collected at baseline and visits at month 1, 3, 6, and 12. The therapeutic target was defined as DAS28-CRP <2.6 in all patients or <3.2 in patients with long disease duration (>24 months). The primary outcome was the percentage of patients with one-year radiographic progression (a change in modified total Sharp score ≥0.5).

**Results:**

Compared with the non-CHB group, a significantly higher percentage of patients with one-year radiographic progression was observed in the CHB group (53% vs. 17%, *p* < 0.001), with smaller proportions of patients achieving therapeutic target at month 6 and month 12 (53% vs. 82% and 53% vs. 75%, both *p* < 0.05), remission at month 6 (DAS28-CRP <2.6, 50% vs. 72%, *p* = 0.039), and American College of Rheumatology (ACR)20/50 responses and good or moderate European League Against Rheumatism (EULAR) responses mainly at month 6 and 12 (all *p* < 0.05). Multivariate logistic regression analysis revealed that CHB status was significantly associated with one-year radiographic progression and failure to achieve therapeutic target within 6 months. HBV reactivation occurred in 34% of patients with CHB during one-year follow up, with two patients suffering hepatitis flare.

**Conclusions:**

HBV infection may play a deleterious role in radiographic and clinical outcomes in patients with RA, and HBV reactivation should be paid close attention during immunosuppressive therapy.

**Electronic supplementary material:**

The online version of this article (10.1186/s13075-018-1548-5) contains supplementary material, which is available to authorized users.

## Background

Rheumatoid arthritis (RA) is a systemic autoimmune disease affecting millions of people worldwide, which is characterized by synovitis with bone and cartilage destruction. The etiology of RA remains largely unknown [1]. Genetic predisposition is not sufficient to explain RA development, and environmental triggers, especially infectious agents such as Epstein-Barr virus, cytomegalovirus, and *Proteus mirablis*, have been reported to be linked with RA pathogenesis [2]. New evidence shows that the oral and gut microbiomes are perturbed in patients with RA and partly normalized after RA treatment, suggesting a significant role of microbiomes in RA [3]. Despite these studies, the identity and pathogenicity of most factors implicating a role in RA are not yet clear.

Hepatitis B virus (HBV) infection continues to be one of worldwide leading disease burdens. About 248 million (3.61%) individuals globally have been reported as HBV surface antigen (HBsAg)-positive, with 74 million Chinese patients (5.49%) in 2010 [4]. HBV primarily infects human hepatocytes, and also leads to a series of extrahepatic manifestations or diseases such as polyarthritis, glomerulonephritis, polyarteritis nodosa, and cryoglobulinemia [5]. HBV-related antigens and nucleic acids have been demonstrated in a variety of extrahepatic tissues, including lymph nodes, kidney, skin, colon, stomach, testis, and ovaries [6]. Only a few studies have revealed a possibility of the presence of HBV proteins and DNA in synovial tissues from patients with HBV infection [7, 8]. Surprisingly, several patients with concurrent HBV infection who fulfilled the diagnostic criteria of American College of Rheumatology (ACR) for RA were shown to have disease resolved by anti-HBV treatment [9, 10]. Additionally, prior studies have documented higher serum HBsAg positivity in patients with RA than in non-RA controls [11, 12]. Our previous studies identified 11.2% HBsAg positivity in Chinese patients with RA and HBV reactivation as a common but tricky complication in HBsAg-positive patients with RA undergoing immunosuppressive therapy [13, 14]. Thus, HBV infection and RA are somehow linked. However, until now, no longitudinal studies have examined the role of HBV infection in disease progression during RA treatment. Therefore, the aim of the present study was to explore the influence of HBV infection on therapeutic response and the safety of immunosuppressive therapy in patients with RA with CHB.

## Methods

### Study patients and design

Patients with RA who fulfilled the 1987 revised criteria of the ACR [15] or the 2010 ACR/European League Against Rheumatism (EULAR) criteria for the classification of RA [16] were retrospectively recruited between June 2012 and August 2016 at Sun Yat-Sen Memorial Hospital from a prospective cohort, as described in our previous study [17]. All patients were treated according to the “treat-to-target” strategy [18, 19] and completed at least one year of follow up. Consecutive patients with RA with HBsAg positivity persisting in serum for more than 6 months were recruited as the CHB group. Each patient with RA in the CHB group was matched by age (± 5 years), sex, and disease activity at baseline to four subjects without HBsAg positivity or HBV DNA in serum, who comprised the non-CHB group. The timing of baseline for the four matched patients in the non-CHB group was as close as possible to baseline for the corresponding patient in the CHB group. Other inclusion criteria were patients aged ≥ 18 years and patients with active disease defined as Disease Activity Score 28-joint assessment (DAS28) with four variables including C-reactive protein (DAS28-CRP) ≥2.6. The exclusion criteria were elevated aminotransferase at baseline; other overlapping autoimmune diseases (e.g. systemic lupus erythematosus, scleroderma, dermatomyositis, polyarteritis nodosa, cryoglobulinaemia, nephritis); Wilson’s disease, steatohepatitis, hemochromatosis, *Schistosomiasis japonica*, or drug-induced hepatitis; concomitant infection with hepatitis C virus, hepatitis D virus, or human immunodeficiency virus; other serious infection, organ dysfunction, or malignancy; and lactating or pregnant women. This study was conducted in compliance with the Helsinki Declaration. Due to the retrospective nature of the study, informed consent was waived. The study was approved by the Medical Ethics Committee of Sun Yat-Sen Memorial Hospital (identifier SYSEC-KY-KS-011).

### Treatment and clinical data collection

All patients were treated according to the 2008/2012 ACR [20, 21] and the 2010/2013 EULAR [18, 19] recommendations for the management of RA. The therapeutic target was defined as DAS28-CRP <2.6 in all patients or <3.2 in patients with long disease duration (>24 months) [18, 19]. Available clinical data on patients with RA were collected in this study at baseline and at visits at months 1, 3, 6, and 12, as described before [17], including 28-joint tender and swollen joint counts (TJC28 and SJC28), patient and provider global assessment of disease activity (PtGA and PrGA, respectively), pain visual analog scale (pain VAS), the Stanford Health Assessment Questionnaire (HAQ), erythrocyte sedimentation rate (ESR), CRP, rheumatoid factor (RF), and anti-cyclic citrullinated peptide antibody (ACPA). Besides DAS28-CRP, disease activity was also assessed using the Simplified Disease Activity Index (SDAI), the Clinical Disease Activity Index (CDAI), and the Routine Assessment of Patient Index Data 3 (RAPID3) [22]. Cumulative doses of oral glucocorticosteroids (GCs) and disease-modifying anti-rheumatic drugs (DMARDs) were recorded during one-year follow up. GC doses were converted to a prednisone-equivalent dose.

Serological markers of HBV infection, including HBsAg, HBV e antigen (HBeAg), the core HBV antigen (HBcAg), and the corresponding antibodies to these antigens (anti-HBs, anti-HBe, and anti-HBc, respectively), were tested in all patients with RA using enzyme-linked immunosorbent assay (Zhongshan Biotechnology CO., LTD, Guangdong, China) or electrochemluminescence-immunoassay (Roche Diagnostics, Mannheim, Germany). HBV DNA was measured using a commercially available quantitative real-time polymerase chain reaction kit (Da An Gene Co., Ltd. of Sun Yat-Sen University, Guangdong, China), with the lowest detection threshold of 500 IU/mL. Liver function including alanine aminotransferase (ALT, U/L, normal range 5–40 U/L) and aspartate transaminase (AST, U/L, normal range 5–40 U/L) was tested at each visit during follow up. HBV serological markers and HBV DNA levels were evaluated in all patients with RA at baseline and every 1–3 months during follow up in the CHB group. These parameters in the non-CHB group were re-examined if aminotransferase was elevated during follow up. Antiviral prophylaxis by entecavir or tenofovir was suggested for all patients with RA with CHB before RA treatment. Moreover, serum levels of soluble matrix metalloproteinase (MMP-3) were tested as described before [23]. The normal ranges of serum MMP-3 concentrations were 18–60 ng/mL (women) and 24–120 ng/mL (men).

### Radiographic assessments

Radiographs of the bilateral hands and wrists (anteroposterior view) of all patients were performed at baseline and month 12. The Sharp/van der Heijde-modified total Sharp score (mTSS), joint erosion (JE), and joint space narrowing (JSN) were scored by two experienced observers (MJD from the Department of Rheumatology and YZH from the Department of Radiology), who were blinded to the patients’ clinical data. As described previously [17], reliability and agreement were assessed based on the intra-class correlation coefficient (ICC): the mean ICC for inter-observer agreement was 0.950. Bony erosion was defined as a cortical break identified on radiography [24]. Radiographic progression was defined as a change in mTSS (ΔmTSS) ≥0.5 units [25], and rapid radiographic progression (RRP) was defined as ΔmTSS ≥5 units from baseline to month 12 [26].

### Outcome assessments

The primary outcome was the percentage of patients with one-year radiographic progression. The secondary outcomes were determined at each visit. These were the percentages of patients achieving therapeutic target and remission, rates of EULAR responses and ACR20/50/70 responses [27], and changes in disease activity indicators.

### Safety assessments

Side effects were recorded and evaluated at each visit. Neutropenia was defined as neutrophil count <2.0 × 10^9^/L. HBV reactivation was defined as the reappearance of HBsAg in a patient with resolved HBV infection, the detection of previously undetectable HBV DNA or > 1 log10 (10-fold) IU/mL increase in serum HBV DNA, and rise in HBV DNA level above an arbitrary cutoff (for example, 20,000 IU) in patients with biochemical worsening [28–30]. A hepatitis flare was determined to be present when ALT was greater than two times the upper limit of the normal range (ULN) [30].

### Statistical analysis

Statistical analyses were performed using IBM SPSS Statistics version 20.0 software (IBM, Armonk, NY, USA). Descriptive statistics (median, interquartile range (IQR) or 5th/95th percentile ranges) were calculated for continuous variables and categorical variables were presented as frequencies and percentages. Conditional logistic regression analysis was used to compare continuous and categorical variables between the CHB group and the non-CHB group, and odds ratios (ORs) and 95% confidence intervals (CIs) were calculated to identify risk factors for one-year radiological progression and failure to achieve therapeutic target within 6 months. Variables were included in the equation when the *p* value was < 0.05 or removed when the *p* value was > 0.10, following the step-forward selection rule. A two tailed *p* value <0.05 was considered statistically significant.

## Results

### Demographic characteristics of patients with RA at baseline

A total of 32 patients with RA with CHB were included in the study, of whom 27 (84%) were female. There were 72% of patients who were both RF and ACPA positive, and 78% of patients had bony erosion at baseline. Eleven (34%) patients were treatment-naïve, without previous GC or DMARD therapy for 6 months before enrollment. Fourteen (44%) patients had a level of HBV DNA above 500 IU/mL, of whom 11 (34%) had levels above 10^3^ IU/mL and 8 (25%) had levels above 10^4^ IU/mL, all with normal liver function. A total of 128 age-matched, sex-matched, and baseline disease activity-matched RA controls in the non-CHB group were compared with patients with RA with CHB. There was no significant difference in baseline demographic and clinical characteristics between groups, except for significantly higher levels of JE subscore and mTSS, and significantly greater proportions of patients using sulfasalazine (SSZ) and hydroxychloroquine (HCQ) in the previous 6 months before enrollment in the CHB group (both *p* < 0.05, Table 1).

**Table 1:** Demographic and disease characteristics at baseline

Parameters	CHB group (*n* = 32)	Non-CHB group (*n* = 128)	*p* value^a^
Matched parameters			
Female, *n* (%)	27 (84)	108 (84)	1.000
Age (years)	49 (38–56)	49 (38–57)	0.964
DAS28-CRP	4.6 (3.5–5.0)	4.6 (3.8–5.3)	0.791
Demographic characteristics			
Age of onset (years)	44 (31–52)	45 (33–53)	0.948
Disease duration (months)	36 (9–113)	36 (12–84)	0.805
Short duration (<6 months)	3 (9)	19 (15)	0.476
Intermediate duration (6–24 months)	12 (38)	39 (30)	0.501
Long duration (>24 months)	17 (53)	70 (55)	0.887
Disease characteristics			
TJC28	6 (2–10)	6 (3–10)	0.914
SJC28	4 (1–8)	4 (2–6)	0.523
PainVAS	4 (2–5)	4 (3–6)	0.131
PtGA	5 (3–6)	5 (3–6)	0.355
PrGA	5 (3–5)	5 (3–6)	0.276
HAQ	0.6 (0–1.3)	0.6(0.1–1.2)	0.888
CRP (mg/L)	12.1 (3.8–44.2)	12.1 (4.8–32.0)	0.543
ESR (mm/h)	36 (20–62)	50 (24–75)	0.162
RF positivity, *n* (%)	23 (72)	96 (75)	0.746
ACPA positivity, *n* (%)	23 (72)	101 (79)	0.448
SDAI	22.1 (13.6–31.3)	21.2 (14.6–29.3)	0.320
CDAI	19 (12–28)	20 (13–26)	0.332
RAPID3	3.3 (2.1–4.6)	3.6 (2.7–5.2)	0.128
MMP-3 (ng/mL)	184 (86–453)	155 (86–358)	0.440
Liver function			
AST (U/L)	18 (14–25)	16 (14–21)	0.630
ALT (U/L)	18 (12–29)	15 (10–21)	0.769
Radiographic status			
Bony erosions, *n* (%)	25 (78)	96 (75)	0.742
JSN subscore	5.5 (0–18.8)	3.0 (1.0–9.8)	0.078
JE subscore	6.0 (1.0–22.0)	4.0 (0.3–10.0)	**0.009**
mTSS	11.0 (1.3–36.3)	8.0 (2.0–20.8)	**0.021**
Previous medications, *n* (%)			
Treatment-naïve^b^	11 (34)	55 (43)	0.431
GCs	14 (44)	48 (38)	0.562
MTX	16 (50)	49 (38)	0.283
LEF	6 (19)	34 (27)	0.417
SSZ	4 (13)	1 (1)	**0.005**
HCQ	10 (31)	12 (9)	**0.006**
CysA	1 (3)	5 (4)	0.853
Biologic agents	1 (3)	3 (2)	0.821

Data are presented as median (interquartile range (IQR)) or number (percentage (%))

*ACPA* anti-cyclic citrullinated peptide antibody, *ALT* alanine aminotransferase, *AST* aspartate transaminase, *CDAI* Clinical Disease Activity Index, *CHB* chronic hepatitis B virus infection, *CRP* C-reactive protein, *CysA* cyclosporin A, *DAS28* Disease Activity Score 28-joint assessment, *DMARD* disease-modifying anti-rheumatic drug, *ESR* erythrocyte sedimentation rate, *GC* glucocorticosteroid, *HAQ* Stanford Health Assessment Questionnaire, *HCQ* hydroxychloroquine, *JE* joint erosion, *JSN* joint space narrowing, *LEF* leflunomide, *mTSS* modified total Sharp score, *MMP-3* matrix metalloproteinase-3, *MTX* methotrexate, *NA* not applicable, *Pain VAS* pain visual analog scale, *PrGA* provider global assessment of disease activity, *PtGA* patient global assessment of disease activity, *RA* rheumatoid arthritis, *RAPID3* Routine Assessment of Patient Index Data 3, *RF* rheumatoid factor, *SDAI* Simplified Disease Activity Index, *SJC28* 28-joint swollen joint counts, *SSZ* sulfasalazine, *TJC28* 28-joint tender joint count

^a^Compared between the CHB group and the non-CHB group using conditional logistic regression analysis: bold *p* values are significant

^b^Without glucocorticosteroid or DMARD therapy for 6 months before enrollment

### Comparison of treatment after enrollment

Initial treatment after enrollment and therapy adjustment were according to the “treat-to-target” strategy and patient’s willingness. There was no significant difference between groups in the initial therapy using GCs, methotrexate (MTX), iguratimod, or biologic agents after enrollment (all *p* > 0.05, Table 2). Compared with the non-CHB group, significantly higher percentages of patients in the CHB group took SSZ and HCQ (44% vs. 2% and 75% vs. 11%, respectively; both *p* < 0.001), while a significantly smaller proportion of patients with CHB took leflunomide (LEF) (16% vs. 84%, *p* < 0.001). Accordingly, patients in the CHB group received significantly higher cumulative doses of SSZ, HCQ, and cyclosporin A (CysA), while taking significantly lower cumulative doses of methotrexate (MTX) and LEF both within the initial 6 months after enrollment and during one-year follow up (all *p* < 0.05). Combined DMARDs were used in 31 (97%) patients in the CHB group and 125 (98%) in the non-CHB group. Compared with the non-CHB group, a greater proportion of CHB patients used the regimen of MTX combined with SSZ and HCQ (34% vs. 1%, *p* < 0.001), while a smaller percentage of patients in the CHB group used the regimen of MTX combined with LEF (9% vs. 72%, *p* < 0.001). Six (19%) patients in the CHB group and 38 (30%) in the non-CHB group were treated with a combination of conventional synthetic DMARDs (csDMARDs) and biologic agents (tocilizumab, infliximab, or recombinant human tumor necrosis factor-α receptor-II (Yi Sai Pu, biosimilar)).

**Table 2:** Medications after enrollment

Medication	CHB group (n = 32)	Non-CHB group (n = 128)	*p* value^a^
Initial medications, *n* (%)			
GCs	23 (72)	89 (70)	0.817
<5 mg/day	1 (3)	3 (2)	0.821
≥5, ≤10 mg/day	19 (59)	81 (63)	0.715
>10, ≤20 mg/day	2 (6)	4 (3)	0.462
>20 mg/day	1 (3)	1 (1)	0.357
MTX	28 (87)	121 (95)	0.217
≤10 mg/week	21 (66)	79 (62)	0.715
>10, ≤15 mg/week	6 (19)	40 (31)	0.217
>15 mg/week	1 (3)	2 (2)	0.606
LEF	5 (16)	107 (84)	**<0.001**
SSZ	14 (44)	2 (2)	**<0.001**
HCQ	24 (75)	14 (11)	**<0.001**
CysA	4 (13)	4 (3)	0.062
Iguratimod	1 (3)	10 (8)	0.415
Biologic agents	6 (19)	38 (30)	0.273
Tocilizumab	4 (13)	30 (23)	0.234
Yi Sai Pu	2 (6)	5 (4)	0.606
Infliximab	0 (0)	3 (2)	NA
Six-month cumulative dose of medications^b^ (mg)			
GCs	900 (0–1406)	1069 (0–1556)	0.506
MTX	260 (201–315)	288 (260–348)	**0.024**
LEF	0 (0–0)	1800 (1500–3263)	**<0.001**
SSZ	0 (0–270,000)	0 (0–0)	**<0.001**
HCQ	54,000 (3000–72,000)	0 (0–0)	**<0.001**
CysA^c^	0 (0–19,515)	0 (0–4500)	**0.020**
Iguratimod^c^	0 (0–2625)	0 (0–9000)	0.330
Tocilizumab^c^	0 (0–1880)	0 (0–2400)	0.218
Yi Sai Pu^c^	0 (0–600)	0 (0–0)	0.606
Infliximab^c^	0 (0–0)	0 (0–0)	NA
One-year cumulative dose of medications (mg)			
GCs	1800 (0–2790)	1744 (0–2475)	0.418
MTX	520 (413–650)	585 (520–715)	**0.023**
LEF	0 (0–0)	3600 (2719–6600)	**<0.001**
SSZ	68,000 (0–536,000)	0 (0–0)	**<0.001**
HCQ	90,000 (3000–144,000)	0 (0–0)	**<0.001**
CysA^c^	0 (0–37,575)	0 (0–12,825)	**0.016**
Iguratimod^c^	0 (0–5775)	0 (0–9000)	0.393
Tocilizumab^c^	0 (0–1880)	0 (0–2800)	0.184
Yi Sai Pu^c^	0 (0–600)	0 (0–0)	0.606
Infliximab^c^	0 (0–0)	0 (0–0)	NA

Data are presented as median (interquartile range (IQR)) or number (percentage (%)) unless stated otherwise

*CHB* chronic hepatitis B virus (HBV) infection, *CysA* cyclosporin A, *GC* glucocorticosteroid, *HCQ* hydroxychloroquine, *LEF* leflunomide, *MTX* methotrexate, *NA* not applicable, *SSZ* sulfasalazine

^a^Compared between the CHB group and the non-CHB group using conditional logistic regression analysis: bold *p* values are significant

^b^Within the initial 6 months after enrollment

^c^Data were analyzed as median (5th/95th percentile ranges) due to the small number of patients using these medications

Antiviral prophylaxis by entecavir or tenofovir was suggested for all patients with RA with CHB. However, due to economic reasons, only 14 (44%) patients finally accepted, of whom 5 (36%) chose lamivudine, 6 (43%) adefovir, and only 3 (21%) entecavir. Notably, there were no significant differences in GC therapy, DMARD therapy, or cumulative doses of the medications after enrollment between patients with and without antiviral prophylaxis. Antiviral therapy was commenced or switched to agents with a high barrier to resistance, such as entecavir or tenofovir, when HBV reactivation occurred. In addition, liver function was closely monitored as frequently as 2 to 4 weeks and GC, MTX, or LEF therapies were tapered or withdrawn, especially if hepatitis flare occurred.

### Patients with RA with CHB had more pronounced radiographic progression at month 12

**Fig. 1:**
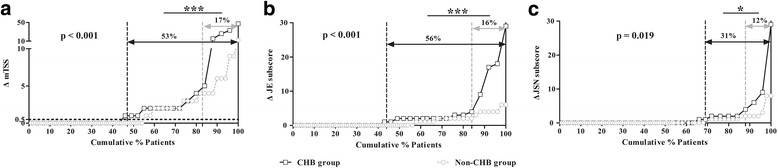
One-year radiographic change in patients with rheumatoid arthritis (RA) in the chronic hepatitis B virus infection (CHB) group and the non-CHB group. Comparison of cumulative probability of △mTSS (**a**), △JSN subscore (**b**) and △JE subscore (**c**) during one-year follow up between the CHB group and the non-CHB group: *p < 0.05, **p < 0.01, ***p < 0.001. JE, joint erosion; JSN, joint space narrowing; mTSS, modified total Sharp score

Compared with the non-CHB group, there was a significantly higher percentage of patients in the CHB group experiencing one-year radiographic progression (53% vs. 17%, *p* < 0.001), with a similar trend in RRP (16% vs. 5%, *p* = 0.059). Significantly higher levels of JE subscore, JSN subscore, and mTSS at month 12 were observed and there were more increases in JE subscore and mTSS in the CHB group than those in the non-CHB group (all *p* < 0.05). The cumulative probability distribution of radiographic change from baseline to month 12 in patients with RA in both groups and the space between the curves indicated there was a significantly higher percentage of patients with CHB who had one-year radiographic progression (Fig. 1a-c).

### Patients with RA with CHB achieved a lower level of clinical response

**Fig. 2:**
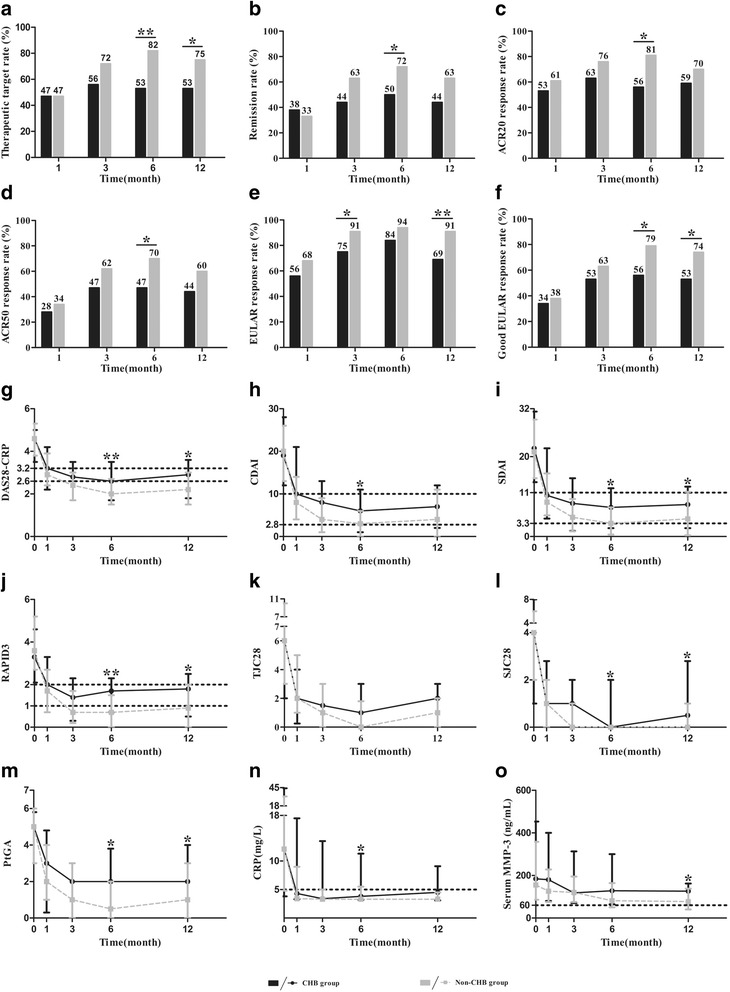
Clinical responses in the chronic hepatitis B virus infection (CHB) group and the non-CHB group. **a-f** Comparison of the rates of patients achieving therapeutic target, remission, American College of Rheumatology (ACR)20/50 responses, and European League Against Rheumatism (EULAR) responses at each visit. **g-n** Comparison of dynamic disease activity indicators at each visit. **o** Comparison of dynamic matrix metalloproteinase-3 (MMP-3) levels in female patients at each visit. Data are represented by the median and interquartile range: *p < 0.05, **p < 0.01, ***p < 0.001. CDAI, Clinical Disease Activity Index; CRP, C-reactive protein; DAS28, Disease Activity Score 28-joint assessment; Pain VAS, pain visual analog scale; PrGA, provider global assessment of disease activity; PtGA, patient global assessment of disease activity; RAPID3, Routine Assessment of Patient Index Data 3; SDAI, Simplified Disease Activity Index; SJC28, 28-joint swollen joint counts; TJC28, 28-joint tender joint counts

At month 6, the proportions of patients achieving therapeutic target (53% vs. 82%, *p* = 0.003) and remission (50% vs. 72%, *p* = 0.039) were significantly smaller in the CHB group than those in the non-CHB group, with a similar trend of achieving therapeutic target and remission at month 12 (53% vs. 75%, *p* = 0.034 and 44% vs. 63%, *p* = 0.077, respectively) (Fig. 2a, b). The percentages of ACR20 and ACR50 responders were also significantly lower in the CHB group versus the non-CHB group at month 6 (56% vs. 81%, *p* = 0.013 and 47% vs. 70%, *p* = 0.029, respectively) (Fig. 2c, d). Additionally, compared with the non-CHB group, the percentage of patients achieving good or moderate EULAR response was significantly lower in the CHB group at month 3 (75% vs. 91%, *p* = 0.038) and month 12 (69% vs. 91%, *p* = 0.004); a significantly smaller proportion of patients achieving a good EULAR response was observed in the CHB group at month 6 (56% vs. 79%, *p* = 0.022) and month 12 (53% and 74%, *p* = 0.041) (Fig. 2e, f).

The results of dynamic disease activity indicators during one-year follow up are shown in Fig. 2g-n. Disease activity indicators, except for ESR, TJC28, HAQ, were significantly higher in the CHB group than those in the control group mainly at month 6 and month 12 (all *p* < 0.05). An additional file shows the other clinical responses that were not demonstrated in Fig. 2 (Additional file 1). Moreover, a significantly higher serum MMP-3 level was observed in female patients from the CHB group compared with that in the non-CHB group at month 12 (*p* = 0.020, Fig. 2o). Regardless of gender, a similar trend in the serum MMP-3 level was also seen at month 6 (*p* = 0.086, Additional file 1).

### Risk factors for one-year radiographic progression and failure to achieve therapeutic target within 6 months

To determine risk factors for one-year radiographic progression, univariate logistic regression analysis was performed on variables including baseline characteristics, CHB status, and RA medications after enrollment (including categories of medications, one-year cumulative doses of medications, and different regimens of combined therapies) (Table 3). The results revealed that CHB status (OR 3.129, 95% CI 1.661–5.895; *p* < 0.001), higher baseline mTSS (OR 1.016, 95% CI 1.006–1.026; *p* = 0.001), and higher one-year cumulative dose of GCs (OR 1.000282, 95% CI 1.000067–1.000497, *p* = 0.010) were significant factors for one-year radiographic progression. In bivariate analyses that adjusted for baseline mTSS and one-year cumulative dose of GCs, one factor at a time, CHB status was still positively associated with one-year radiographic progression (OR 2.610 and OR 2.881, respectively; both *p* < 0.01). Moreover, multivariate logistic regression analysis that adjusted for all significant factors from univariate analyses showed that CHB status was independently associated with one-year radiographic progression (OR 2.403, 95% CI 1.218–4.743; *p* = 0.011).

**Table 3:** Logistic regression analyses of risk factors for one-year radiographic progression

	OR	95% CI	*p* value*
Univariate analyses			
Female	0.845	(0.373–1.915)	0.687
Age	0.993	(0.969–1.018)	0.598
Disease duration	1.004	(1.000–1.008)	0.076
CHB status	3.129	(1.661–5.895)	**<0.001**
TJC28	0.996	(0.934–1.063)	0.914
SJC28	0.982	(0.908–1.062)	0.651
Pain VAS	1.056	(0.905–1.232)	0.491
PtGA	1.094	(0.939–1.274)	0.248
PrGA	1.086	(0.921–1.281)	0.326
HAQ	1.346	(0.990–1.830)	0.058
CRP	1.001	(0.998–1.014)	0.876
ESR	0.999	(0.989–1.008)	0.786
RF positivity	2.355	(0.921–6.023)	0.074
ACPA positivity	1.983	(0.775–5.070)	0.153
DAS28-CRP	1.011	(0.742–1.378)	0.944
MMP-3	1.001	(0.999–1.002)	0.292
mTSS	1.016	(1.006–1.026)	**0.001**
Treatment-naïve^b^	0.710	(0.365–1.381)	0.313
GCs	2.300	(0.964–5.489)	0.061
MTX	1.369	(0.330–5.678)	0.666
LEF	0.684	(0.359–1.304)	0.248
SSZ	1.984	(0.876–4.495)	0.101
HCQ	1.611	(0.828–3.135)	0.160
CysA	1.591	(0.490–5.167)	0.440
Iguratimod	1.130	(0.348–3.670)	0.839
Biologic agents	0.477	(0.200–1.139)	0.095
MTX combined with SSZ and HCQ	2.265	(0.949–5.406)	0.066
MTX combined with LEF	0.719	(0.384–1.346)	0.302
One-year cumulative dose of GCs^c^	1.000	(1.000–1.000)	**0.010**
One-year cumulative dose of MTX	0.999	(0.998–1.000)	0.224
One-year cumulative dose of LEF^d^	1.000	(1.000–1.000)	0.074
One-year cumulative dose of SSZ	1.001	(1.000–1.003)	0.133
One-year cumulative dose of HCQ	1.004	(0.998–1.009)	0.160
One-year cumulative dose of CysA^e^	1.000	(1.000–1.000)	0.124
One-year cumulative dose of iguratimod^f^	1.000	(1.000–1.000)	0.761
One-year cumulative dose of tocilizumab^g^	1.000	(0.999–1.000)	0.232
One-year cumulative dose of Yi Sai Pu	1.000	(0.998–1.003)	0.817
One-year cumulative dose of infliximab	0.997	(0.989–1.006)	0.564
Bivariate models			
CHB status adjusted for baseline mTSS	2.610	(1.338–5.092)	**0.005**
CHB status adjusted for one-year cumulative dose of GCs	2.881	(1.511–5.493)	**0.001**
Multivariate model			
CHB status adjusted for baseline mTSS and one-year cumulative dose of GCs	2.403	(1.218–4.743)	**0.011**

*ACPA* anti-cyclic citrullinated peptide antibody, *CHB* chronic hepatitis B virus (HBV) infection, *CI* confidence interval, *CRP* C-reactive protein, *CysA* cyclosporin A, *DAS28* Disease Activity Score 28-joint assessment, *ESR* erythrocyte sedimentation rate, *GC* glucocorticosteroid, *HAQ* Stanford Health Assessment Questionnaire, *HCQ* hydroxychloroquine, *LEF* leflunomide, *mTSS* modified total Sharp score, *MTX* methotrexate, *OR* odds ratio, *Pain VAS* pain visual analog scale, *PrGA* provider global assessment of disease activity, *PtGA* patient global assessment of disease activity, *RF* rheumatoid factor, *SJC28* 28-joint swollen joint count, *SSZ* sulfasalazine, *TJC28* 28-joint tender joint count

^a^Calculated using conditional logistic regression analysis. Univariate logistic regression analysis was performed on variables, including baseline characteristics, CHB status, and rheumatoid arthritis medications after enrollment (including categories of medications, one-year cumulative doses of medications, and different regimens of combined therapies); bivariate analysis was performed by adjusting for baseline mTSS and one-year cumulative dose of GCs respectively; multivariate logistic regression analysis was performed by adjusting for all significant univariate factors: bold *p* values are significant

^b^Without glucocorticosteroid or DMARD therapy for 6 months before enrollment

^c^One-year cumulative dose of GCs: OR 1.000282, 95% CI 1.000067–1.000497; *p* = 0.010

^d^One-year cumulative dose of LEF: OR 0.999888, 95% CI 0.999766–1.000011; *p* = 0.074

^e^One-year cumulative dose of CysA: OR 1.000025, 95% CI 0.999993–1.000057; *p* = 0.124

^f^One-year cumulative dose of iguratimod: OR 1.000013, 95% CI 0.999926–1.000101; *p* = 0.761

^g^One-year cumulative dose of tocilizumab: OR 0.999719, 95% CI 0.999259–1.000180; *p* = 0.232

Both ACR and EULAR recommendations for the management of RA emphasize tight control of disease activity and attaining therapeutic target within 6 months [19, 21]. Therefore, logistic regression analysis was also performed to determine risk factors for failure to achieve therapeutic target within 6 months (Table 4). The results of univariate logistic regression analysis revealed that CHB status (OR 3.077, 95% CI 1.349–7.017; *p* = 0.008), being medication-naïve (OR 0.300, 95% CI 0.102–0.881; *p* = 0.029), MTX therapy (OR 0.266, 95% CI 0.099–0.716; *p* = 0.009), a regimen of MTX combined with LEF (OR 0.365, 95% CI 0.155–0.861; *p* = 0.021), 6-month cumulative dose of MTX (OR 0.997, 95% CI 0.993–1.000; *p* = 0.049), and 6-month cumulative dose of CysA (OR 1.000077, 95% CI 1.000011–1.000143; *p* = 0.023) were recognized as significant factors for failure to achieve therapeutic target within 6 months. Bivariate analyses that adjusted for the significant factors in the univariate analyses, one factor at a time, demonstrated that CHB status was always an independent risk factor (OR 2.722–3.077, *p* = 0.019–0.008). Further multivariate logistic regression analysis was performed by adjusting for all significant factors in the univariate analyses. Due to the multicollinearity between MTX therapy and 6-month cumulative dose of MTX, two multivariate models were set up respectively and the results of both models showed that CHB status remained significantly associated with failure to achieve therapeutic target within 6 months (MTX therapy model, OR 2.617, 95% CI 1.140–6.007, *p* = 0.023; 6-month cumulative dose of MTX model, OR 2.844, 95% CI 1.245–6.498, *p* = 0.013).

**Table 4:** Logistic regression analyses of risk factors for failure to achieve therapeutic target within 6 months

	OR	95% CI	*p* value^a^
Univariate analyses			
Female	1.235	(0.367–4.155)	0.734
Age	1.012	(0.979–1.045)	0.488
Disease duration	1.000	(0.994–1.007)	0.969
CHB status	3.077	(1.349–7.017)	**0.008**
TJC28	1.059	0.986–1.137	0.115
SJC28	1.035	0.951–1.127	0.421
Pain VAS	1.096	0.897–1.338	0.369
PtGA	1.192	0.977–1.455	0.083
PrGA	1.142	0.920–1.418	0.230
HAQ	1.318	0.880–1.975	0.180
CRP	1.004	(0.988–1.020)	0.616
ESR	1.000	(0.987–1.021)	0.949
RF positivity	1.637	(0.557–4.811)	0.371
ACPA positivity	0.452	(0.195–1.043)	0.063
DAS28-CRP	1.394	(0.936–2.077)	0.102
MMP-3	1.000	(0.999–1.002)	0.751
mTSS	1.010	(0.995–1.024)	0.195
Treatment-naïve^b^	0.300	(0.102–0.881)	**0.029**
GCs	1.178	(0.465–2.989)	0.729
MTX	0.266	(0.099–0.716)	**0.009**
LEF	0.468	(0.206–1.060)	0.069
SSZ	1.350	(0.401–4.543)	0.628
HCQ	2.064	(0.893–4.768)	0.090
CysA	2.850	(0.847–9.591)	0.091
Iguratimod	2.032	(0.604–6.837)	0.252
Biologic agents	0.555	(0.189–1.631)	0.285
MTX combined with SSZ and HCQ	1.175	(0.275–5.009)	0.828
MTX combined with LEF	0.365	(0.155–0.861)	**0.021**
Six-month cumulative dose of GCs^c^	1.000	(1.000–1.001)	0.340
Six-month cumulative dose of MTX	0.997	(0.993–1.000)	**0.049**
Six-month cumulative dose of LEF^d^	1.000	(0.999–1.000)	0.113
Six-month cumulative dose of SSZ	1.000	(0.995–1.005)	0.890
Six-month cumulative dose of HCQ	1.008	(0.994–1.022)	0.245
Six-month cumulative dose of CysA^e^	1.000	(1.000–1.000)	**0.023**
Six-month cumulative dose of iguratimod^f^	1.000	(1.000–1.000)	0.259
Six-month cumulative dose of tocilizumab^g^	1.000	(0.999–1.000)	0.337
Six-month cumulative dose of Yi Sai Pu	1.001	(0.999–1.004)	0.322
Six-month cumulative dose of infliximab	0.997	(0.986–1.009)	0.659
Bivariate models			
CHB status adjusted for treatment-naïve status	2.844	(1.245–6.498)	**0.013**
CHB status adjusted for MTX	2.722	(1.177–6.298)	**0.019**
CHB status adjusted for the regimen of MTX combined with LEF	3.077	(1.349–7.017)	**0.008**
CHB status adjusted for 6-month cumulative dose of MTX	3.077	(1.349–7.017)	**0.008**
CHB status adjusted for 6-month cumulative dose of CysA	3.077	(1.349–7.017)	**0.008**
Multivariate models			
CHB status adjusted for treatment-naïve status, MTX therapy, the regimen of MTX combined with LEF, and 6-month cumulative dose of CysA	2.617	(1.140–6.007)	**0.023**
CHB status adjusted for treatment-naïve status, 6-month cumulative dose of MTX, the regimen of MTX combined with LEF, and 6-month cumulative dose of CysA	2.844	(1.245–6.498)	**0.013**

*ACPA* anti-cyclic citrullinated peptide antibody, *CHB* chronic hepatitis B (HBV) infection, *CI* confidence interval, *CRP* C-reactive protein, *CysA* cyclosporin A, *DAS28* Disease Activity Score 28-joint assessment, *ESR* erythrocyte sedimentation rate, *GCs* glucocorticosteroids, *HAQ* Stanford Health Assessment Questionnaire, *HCQ* hydroxychloroquine, *LEF* leflunomide, *mTSS* modified total Sharp score, *MTX* methotrexate, *OR* odds ratio, *Pain VAS* pain visual analogue scale, *PrGA* provider global assessment of disease activity, *PtGA* patient global assessment of disease activity, *RF* rheumatoid factor, *SJC28* 28-joint swollen joint count, *SSZ* sulfasalazine, *TJC28* 28-joint tender joint count

^a^Calculated using conditional logistic regression analysis: univariate logistic regression analysis was performed on variables, including baseline characteristics, CHB status, and rheumatoid arthritis medications after enrollment (including categories of medications, 6-month cumulative doses of medications, and different regimens of combined therapies); bivariate analysis was performed by adjusting for the significant univariate factors individually; due to the multicollinearity between MTX therapy and 6-month cumulative dose of MTX, two multivariate models (MTX therapy model and 6-month cumulative dose of MTX model) were set up respectively by adjusting for all significant univariate factors: bold *p* values are significant

^b^Without glucocorticosteroid or DMARD therapy for 6 months before enrollment

^c^Six-month cumulative dose of GCs: OR 1.000220, 95% CI 0.999768–1.000671; *p* = 0.340

^d^Six-month cumulative dose of LEF: OR 0.999738, 95% CI 0.999414–1.000062; *p* = 0.113

^e^Six-month cumulative dose of CysA: OR 1.000077, 95% CI 1.000011–1.000143; *p* = 0.023

^f^Six-month cumulative dose of iguratimod: OR 1.000080, 95% CI 0.999941–1.000218; *p* = 0.259

^g^Six-month cumulative dose of tocilizumab: OR 0.999656, 95% CI 0.998954–1.000358; *p* = 0.337

### Safety

Table 5 presents an overview of side effects during one-year follow up. Infections occurred in 16% of patients with CHB and 13% of patients in the non-CHB group. The most common infections were respiratory tract infection, with one patient (3%) in the CHB group and six paitents (5%) in the control group diagnosed as pneumonia respectively. One patient in the non-CHB group suffered from herpes zoster 7 months after initiation with tocilizumab. There was no significant difference between groups in the incidence of infections, trichomadesis, neutropenia, or gastrointestinal discomfort (all *p* > 0.05). No deaths occurred due to these side effects in this study.

**Table 5:** Safety profile of rheumatoid arthritis treatment

Side effect	CHB group (n = 32)	Non-CHB group (n = 128)	*p* value^a^
Total side effects, *n* (%)	15 (47)	68 (53)	0.572
Infections	5 (16)	16 (13)	0.676
Gastrointestinal discomfort	7 (22)	22 (17)	0.583
Trichomadesis	2 (6)	13 (10)	0.547
Neutropenia	2 (6)	14 (11)	0.484
Aminotransferase elevation	6 (19)	39 (30)	0.244
< two fold ULN	4 (13)	30 (23)	0.234
≥ two fold, < three fold ULN	1 (3)	7 (6)	0.630
≥ three fold ULN	1 (3)	2 (2)	0.606
Antiviral prophylaxis	14 (44)	0 (0)	NA
HBV reactivation	11 (34)	0 (0)	NA
Antiviral prophylaxis (+)	3 (9)	0 (0)	NA
Antiviral prophylaxis (−)	8 (25)	0 (0)	NA

*CHB* chronic HBV infection, *HBV* hepatitis B virus, *NA* not applicable, *ULN* upper limit of the normal range

^a^Compared between the CHB group and the non-CHB group using conditional logistic regression analysis

The main side effects of RA treatment on HBV infection were HBV reactivation and aminotransferase elevation. No patient in the non-CHB group developed HBV reactivation. However, 34% of CHB patients developed virus reactivation, of whom 8 (72%) refused to accept antiviral prophylaxis, with hepatitis flare occurring in 2 patients (6%) during follow up. There was no significant difference in the incidence of aminotransferase elevation between groups (19% vs. 31%, *p* > 0.05). A flowchart of HBV reactivation and hepatitis flare occurring in patients with CHB is shown in Additional file 2. Fortunately, serum HBV DNA level and aminotransferase in all patients with RA returned to undetectable or normal after commencing antiviral therapy or adjusting GC, MTX, or LEF therapy. No liver cirrhosis, liver failure, or HBV-related deaths occurred during follow up in this study.

## Discussion

In this study, we performed a retrospective case-control study to investigate the influence of HBV infection on therapeutic response among patients with RA in a clinical practice setting. The Sharp/van der Heijde score showed that patients with RA with CHB suffered more pronounced one-year radiographic progression than the non-CHB group. Compared with the control group, smaller proportions of patients with RA with CHB achieved therapeutic target and remission, and the trend was also seen in attaining ACR20/50 responses and good or moderate EULAR responses, mainly at month 6 and month 12. Conditional logistic regression analysis showed that CHB status was independently associated with one-year radiographic progression. Both ACR and EULAR recommendations for the management of RA emphasize that therapeutic target should be attained within 6 months [19, 21]. Thus, conditional logistic regression analysis was further performed and the result showed that CHB status was also significantly associated with failure to achieve therapeutic target within 6 months. Therefore, HBV infection may play a deleterious role in radiographic and clinical outcomes in patients with RA. To our knowledge, this is the first study to investigate the role of HBV infection in therapeutic response among patients with RA in clinical practice, which revealed that HBV infection is implicated in RA progression.

HBV infection was complicated due to extrahepatic manifestations including polyarthritis mainly affecting small joints, which seemed to mimic that of  RA and was considered to be immune-complex mediated [31]. Use of recombinant HBsAg could induce the appearance of RA-like symptoms [32, 33], which might result from the common HLA-DR haplotypes for RA [34]. Some patients with HBV infection who fulfilled the 1987 ACR criteria for RA could have symptoms resolved by anti-HBV treatment [9, 10]. Furthermore, a recent large study from Taiwan demonstrated that patients with RA had a higher HBV period prevalence than did the non-RA subjects [11]. Results in the present study showed that patients with RA with CHB had more pronounced one-year radiographic progression and achieved a lower level of clinical response than the non-CHB group, which was in accordance with most previously proposed hypotheses of HBV infection acting as an adverse factor in RA.

Previous studies have shown that even though some patients with HBV infection may have long-lasting polyarthritis, joint destruction remains an almost rare complication. However, a case report describes a patient with positive HBsAg and HBeAg in serum, who suffered worsened pain after a steroid injection for knee osteoarthritis. Radiographs on admission showed a large bone defect in the medial tibia and slight narrowing of the articular gap. Further analysis revealed diffuse expression of HBsAg in the synovium, suggesting destructive knee arthropathy possibly caused by HBV infection [7]. Another report demonstrated that a woman with acute RA onset after receiving the first dose of HBV vaccine experienced erosions with minimal periarticular osteoporosis 10 months later [35]. In this study, a larger proportion of patients with CHB suffered one-year radiographic progression, which implies that HBV infection might directly or indirectly contribute to joint damage. On the other hand, MMP-3 is a proteinase secreted by synovial fibroblasts and chondrocytes in joints, which can accelerate joint destruction in RA. Recently, the reduction in serum MMP-3 was considered as a possible therapeutic target together with disease activity. Urata et al. reported that treating to target MMP-3 normalization combined with disease activity yielded better effects than each target alone in patients with RA [36].

Our previous studies also have shown that serum MMP-3 level was positively correlated with disease activity and continuously elevated serum MMP-3 for 3 to 6 months could predict one-year radiographic progression [17, 23]. In this study, compared with the non-CHB group, serum MMP-3 level was higher in the CHB group at month 6, and was significantly higher in women from the CHB group at month 12, which further confirmed that patients with RA with CHB had a lower level of clinical response and radiographic outcome. Moreover, studies have revealed that HBV X protein could promote cell migration by inducing the transcription, translation, and secretion of MMP-3 [37]. In this scenario, it is possible that CHB status may negatively affect the radiographic and clinical outcomes of patients with RA partly through upregulating MMP-3. Both ACR and EULAR recommendations for the management of RA emphasize tight control of disease activity and therapeutic target should be attained within 6 months [19, 21]. Patients with CHB in this study had more pronounced one-year radiographic progression probably due to a lower level of clinical response during one-year follow up, especially within 6 months. That is, HBV infection may act as a “regulatory factor” or even a “driver factor” during disease progression in patients with RA with CHB. Therefore, it could be speculated that patients with CHB could be classified as having a specific phenotype of RA that may need adjusted regimens or routes of administration to achieve sufficient therapeutic response.

HBV reactivation is a well-recognized complication that may cause high morbidity and mortality in patients who undergo immunosuppressive therapy. The 2008 ACR recommended that SSZ under antiviral prophylaxis and HCQ could be used for patients with RA with CHB and liver function of Child-Pugh class A, while MTX and LEF were contraindicated for all Child-Pugh classifications [19, 21]. However, the guidelines for antiviral prophylaxis made by the American Gastroenterological Association in 2015 recommended low-dose GC therapy (prednisone (or equivalent) at < 10 mg/day) for > 4 weeks in the moderate-risk group and MTX in the low-risk group for HBV reactivation in patients with CHB [30]. LEF is not recommended in patients with a history of hepatitis and might increase the risk of HBV reactivation [14, 38]. In the present study, although not all patients with CHB agreed to receive recommended antiviral prophylaxis due to economic reasons, there was no significant difference in RA treatment between patients with and without antiviral prophylaxis. However, there were significant differences in the medications taken by patients with and without CHB. Patients with RA with CHB were more likely to use SSZ and HCQ rather than LEF, and received significantly lower cumulative doses of MTX. Accordingly, a significantly greater proportion of patients with CHB used the regimen of MTX combined with SSZ and HCQ, while a smaller percentage of patients with CHB used the regimen of MTX combined with LEF. Nevertheless, patients with CHB had a significantly higher possibility of HBV reactivation compared to the non-CHB group (including patients with resolved HBV infection or patients never infected with HBV), which may preclude escalation of therapy and might result in not reaching a full dose of some of the drugs and not achieving therapeutic target within the recommended time frame.

Multivariate logistic regression analysis revealed that both CHB status and MTX therapy were independently associated with failure to achieve therapeutic target within 6 months. Hence, it is reasonable to speculate that patients with CHB had more pronounced one-year radiographic progression and a lower level of clinical response probably due to both CHB status and different regimens, especially MTX therapy, used in the patients with CHB. In light of the pros and cons, it is indeed difficult to find a balance during RA treatment. Therefore, besides CHB status, regimen adjustments for preventing HBV reactivation during RA treatment might also explain part of the adverse role of HBV infection in RA radiographic and clinical outcomes. On the other hand, baseline radiographic status is an important feature that could influence radiographic progression in patients with RA. In this study, levels of JE subscore and mTSS at baseline were significantly higher in the CHB group than in the non-CHB group, and baseline mTSS was one of the significant factors for one-year radiographic progression according to the results of logistic regression analysis. However, CHB status was independently associated with one-year radiographic progression after adjusted for all confounding factors including baseline mTSS.

There are several limitations in this study. First, it was a real-world observational study from a single center and patients were treated with different medications. Although multivariate logistic regression was performed to remove the confounding effect of different medications, it would be necessary to carry out further multicenter studies and balance the combined therapy between groups. Second, over 70% of patients suffered bony erosion at baseline in this study, which was a risk factor for one-year radiographic progression. Hence, more patients with early RA without baseline bony erosion are needed in future to confirm our results. Third, the relatively small number of patients with RA with CHB in this study precluded a robust conclusion. Whether clearance of HBV would lead to a good therapeutic response in RA remains to be clarified in a larger, placebo-controlled study.

## Conclusions

Our results showed that patients with RA with CHB had more pronounced one-year radiographic progression and achieved a lower level of clinical response than those without CHB. HBV reactivation remained a tricky issue in patients with CHB during RA treatment. Thus, HBV infection may play a deleterious role in radiographic and clinical outcomes among patients with RA, and HBV reactivation should be paid close attention during immunosuppressive therapy.

## Additional files

Additional file 1: The other clinical responses between the CHB group and the non-CHB group. Comparison of the other clinical responses at each visit, including ACR70, PrGA, Pain VAS, HAQ, ESR, and serum MMP-3 levels. ACR: American College of Rheumatology; CHB: chronic HBV infection; ESR: erythrocyte sedimentation rate; HAQ: Stanford Health Assessment Questionnaire; MMP-3: matrix metalloproteinase-3; Pain VAS: pain visual analog scale; PrGA: provider global assessment of disease activity. (PDF 1468 kb)

Additional file 2: A flowchart of HBV reactivation occurring in patients with RA with CHB showing antiviral prophylaxis, HBV reactivation, and hepatitis flare in these patients during one-year follow up. CHB: chronic HBV infection; HBV: hepatitis B virus; RA: rheumatoid arthritis. (PDF 33 kb)

## Abbreviations

ACPA: Anti-cyclic citrullinated peptide antibody
ACR: American College of Rheumatology
ALT: Alanine aminotransferase
AST: Aspartate transaminase
CDAI: Clinical Disease Activity Index
CHB: Chronic hepatitis B virus infection
CI: Confidence interval
CRP: C-reactive protein
CysA: Cyclosporin A
DAS28: Disease Activity Score 28-joint assessment
DMARD: Disease-modifying anti-rheumatic drug
ESR: Erythrocyte sedimentation rate
EULAR: European League Against Rheumatism
GC: Glucocorticosteroid
HAQ: Stanford Health Assessment Questionnaire
HBcAg: Hepatitis B virus core antigen
HBeAg: Hepatitis B virus e antigen
HBsAg: Hepatitis B virus surface antigen
HBV: Hepatitis B virus
HCQ: Hydroxychloroquine
IQR: Interquartile range
JE: Joint erosion
JSN: Joint space narrowing
LEF: Leflunomide
MMP-3: Matrix metalloproteinase-3
mTSS: Modified total Sharp score
MTX: Methotrexate
NA: Not applicable
OR: Odds ratio
Pain VAS: Pain visual analog scale
PrGA: Provider global assessment of disease activity
PtGA: Patient global assessment of disease activity
RA: Rheumatoid arthritis
RAPID3: Routine Assessment of Patient Index Data 3
RF: Rheumatoid factor
RRP: Rapid radiographic progression
SDAI: Simplified Disease Activity Index
SJC28: 28-Joint swollen joint counts
SSZ: Sulfasalazine
TJC28: 28-Joint tender joint counts
ULN: Upper limit of the normal range
