# Supplementary figures and images for: Deleterious role of hepatitis B virus infection in therapeutic response among patients with rheumatoid arthritis in a clinical practice setting: a case-control study
Source: Arthritis Res Ther. 2018 May 2;20:81. doi: 10.1186/s13075-018-1548-5 (PMC5932798; doi:10.1186/s13075-018-1548-5)

A

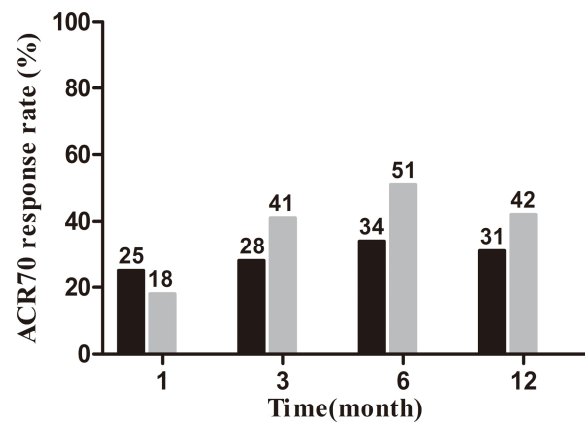

B

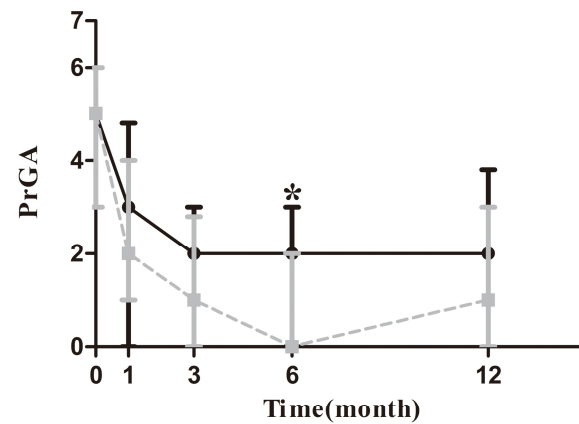

C

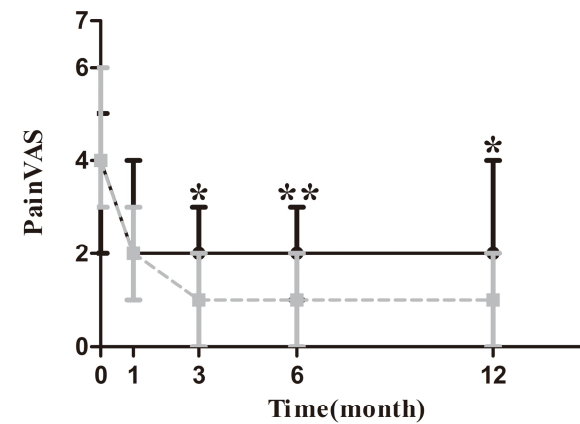

D

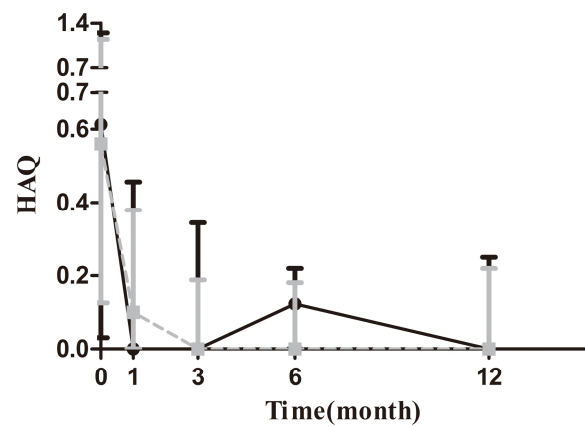

E

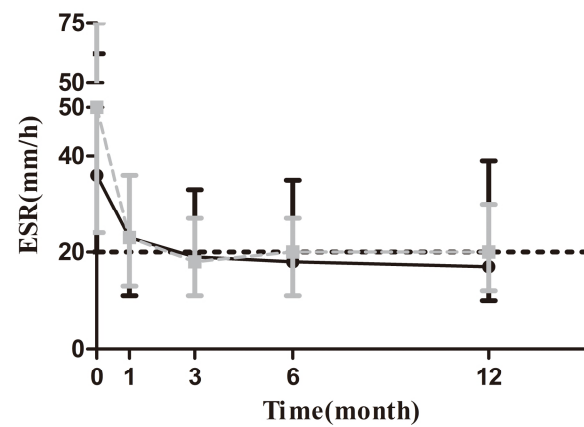

F

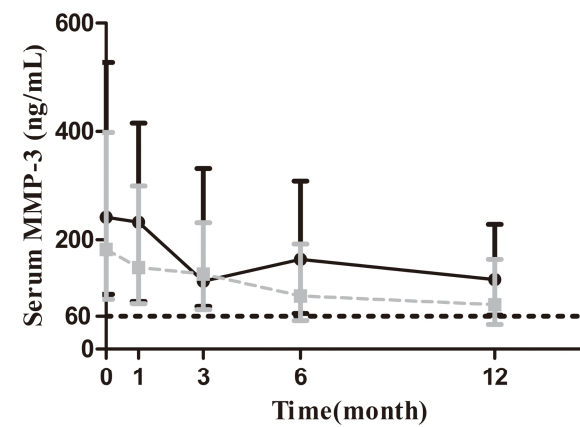

■ / ● CHB group      ■ / □ Non-CHB group

Supplement: Supplementary file 1 — The other clinical responses between the CHB group and the non-CHB group. Comparison of the other clinical responses at each visit, including ACR70, PrGA, Pain VAS, HAQ, ESR, and serum MMP-3 levels. ACR: American College of Rheumatology; CHB: chronic HBV infection; ESR: erythrocyte sedimentation rate; HAQ: Stanford Health Assessment Questionnaire; MMP-3: matrix metalloproteinase-3; Pain VAS: pain visual analog scale; PrGA: provider global assessment of disease activity. (PDF 1468 kb) [file 13075_2018_1548_MOESM1_ESM.pdf]

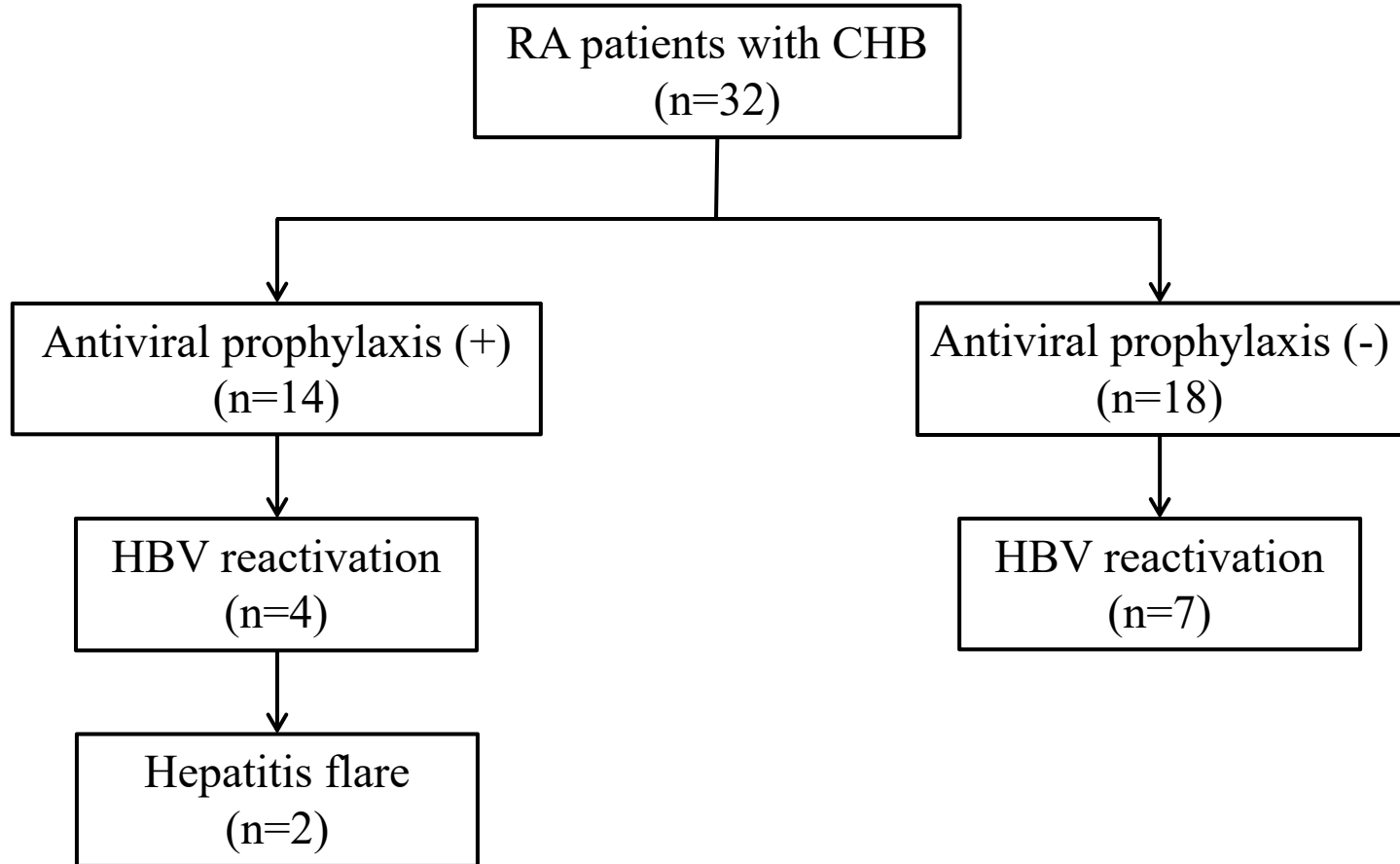

Supplement: Supplementary file 2 — A flowchart of HBV reactivation occurring in patients with RA with CHB showing antiviral prophylaxis, HBV reactivation, and hepatitis flare in these patients during one-year follow up. CHB: chronic HBV infection; HBV: hepatitis B virus; RA: rheumatoid arthritis. (PDF 33 kb) [file 13075_2018_1548_MOESM2_ESM.pdf]
